# The Effect of Applied Hydrostatic Pressures in Ferromagnetic Ordered HoM_2_ [M = (Al, Ni)] Laves Phases: A DFT Study

**DOI:** 10.3390/ma18245510

**Published:** 2025-12-08

**Authors:** Tomás López-Solenzal, David Ríos-Jara, Manuel Ramos, César Fidel Sánchez-Valdés

**Affiliations:** 1 Departamento de Física y Matemáticas, Instituto de Ingeniería y Tecnología, Universidad Autónoma de Ciudad Juárez, 450N Avenida del Charro, Ciudad Juárez 32310, CH, Mexico; al228176@alumnos.uacj.mx (T.L.-S.); manuel.ramos@uacj.mx (M.R.); 2 Instituto Potosino de Investigación Científica y Tecnológica A.C., Camino a la Presa San José 2055, Col. Lomas 4^a^ Sección, San Luis Potosí 78216, SLP, Mexico; david.rios@ipicyt.edu.mx

**Keywords:** HoNi_2_ and HoAl_2_ Laves phases, density functional theory (DFT), Hubbard U correction calculations (DFT+U), hydrostatic pressure effect on magnetic properties

## Abstract

In this study, density functional theory (DFT) with Hubbard U correction calculations (DFT+U) was used to examine the ferromagnetic properties of HoM_2_ Laves phases (M = {Al, Ni}) under external hydrostatic pressure from 0 GPa to 1.0 GPa. The resulting net magnetic moments of 8.61 µ_B_/f.u. (HoAl_2_) and 8.12 µ_B_/f.u. (HoNi_2_) align with values reported in experiments. Additionally, for both alloys, the ferromagnetic behavior remains unchanged under applied pressures from 0 GPa to 1.0 GPa. The study also confirms that the magnetic properties of the alloys are mainly influenced by the 4*f* electrons, with 3*d* electrons playing a slightly more significant role in HoNi_2_ Laves phases compared to HoAl_2_. The contribution of electrons in *d* and *f* orbitals to the net magnetic moment of each Laves phase alloy within the specified pressure range was examined. Furthermore, the crystal geometry optimization and electronic specific heat coefficient were calculated as functions of applied pressures up to 1.0 GPa for both ferromagnetically ordered Laves phases.

## 1. Introduction

Experimental and theoretical research in the field of magnetocaloric-based cooling has grown over the past 25 years, driven by the higher energy efficiency and environmentally friendly nature of this cooling technology compared to the conventional one based on the expansion and compression of gases [1,2,3,4,5,6]. In the last few years, interest in developing magnetic refrigerators for hydrogen liquefaction has encouraged important efforts to find, synthesize, and assess the magnetocaloric response of many families of rare-earth (R)-based alloys due to the significant magnetocaloric response of many compounds below the precooled reference temperature of 77 K [7,8,9,10,11]. Among all the alloy systems investigated, the cubic Laves phases in the RM_2_ systems with M = {Al, Ni} stand out due to the remarkable magnetocaloric properties, especially for heavy R elements such as Ho and Er [6,7,8,9,10,11,12].

These compounds crystallize into the MgCu_2_-type structure (C15, space group: Fd–3m) [12,13], with a lattice parameter of 7.810 Å for HoAl_2_ and 7.130 Å for HoNi_2_ [14,15], and show Weiss–Curie temperatures *T*_C_ of 29 K and 13.4 K, respectively [16,17]. The Laves phases (AB_2_) have two lattice-related characteristics: the relationship between the atomic radii of A and B atoms is between 1.05 and 1.68, and for an atomic radius ratio (r_A_/r_B_) of 1.225, the crystal structures have a higher packing density (around 71%) [12,13,18,19,20,21]. A green hydrogen economy needs hydrogen storage (due to hydriding properties) in the cell unit of Laves C15 phases. Laves intermetallics can be used to store hydrogen interstitially by offering different positions (i.e., three tetrahedral interstices) [13].

Furthermore, HoAl_2_ exhibits magnetic anisotropy, characterized by the easy magnetization axis being <110> for temperatures below 20 K; above this temperature, the easy axis shifts to the <100> intermediate direction [16,22]. Additionally, a spin reorientation occurs at *T*_SR_ = 20 K. The hard magnetization axis in HoAl_2_ is <111> crystal direction [17,22]. The directions of the easy, intermediate, and hard magnetization axes of HoNi_2_ are <100>, <110>, and <111>, respectively [15,22,23,24,25].

On the other hand, density functional theory (DFT) is a fundamental computational method in materials science, providing atomistic insights into crystals and molecules. Resolving a material’s electronic structure enables the investigation and prediction of the structure–property relationships and the underlying physicochemical phenomena in solids [26,27,28,29,30].

The present work investigates, through density functional theory with Hubbard U correction calculations (DFT+U), the effect of hydrostatic pressure on the electronic and magnetic properties of the ferromagnetically ordered HoAl_2_ and HoNi_2_ Laves phases. The spin polarization calculations are performed along the <001> crystal direction. We systematically explore how the electronic density of states is affected in the HoAl_2_ and HoNi_2_ alloys. The theoretical calculations indicate significant changes in the electronic structure under a small hydrostatic pressure of 0.1 GPa in HoNi_2_. A multicaloric approach in the solid-state cooling technology based on the magnetocaloric and barocaloric effects has previously been used [2,3]. The combination of different external fields (e.g., magnetic field and hydrostatic pressure) enables an enhancement of the caloric response by tailoring the magnetic moment (related to the electronic density of states) with the pressure during the magnetic phase transition. Our finding is that the net magnetic moment is modified (drops by 22.5%) when a 0.1 GPa hydrostatic pressure is applied. To our knowledge, the electronic and magnetic properties of HoAl_2_ and HoNi_2_ under hydrostatic pressure, as determined by ab initio calculations, have not yet been reported.

## 2. Materials and Methods

The present study was performed using the Cambridge Serial Total Energy Package (CASTEP) within the density functional theory framework using BIOVIA Materials Studio^®^ 2020 (version 20.1.0.5). For the exchange correlation, the revised Perdew–Burke–Ernzerhof (RPBE) functional was applied as part of the generalized gradient approximation (GGA). It is known that the GGA method fails to correctly describe the localized 4*f* and 3*d* electrons; therefore, the DFT+U (U-Hubbard) correction was introduced into the calculations [31,32,33]. It is important to note that U corrections within GGA show better accuracy when investigating the magnetic and electronic structures of 4*f* and 3*d* compounds (e.g., strongly correlated systems) compared with local density approximation (LDA) or hybrid functionals [34,35]. The U values of localized electrons were 2.50 eV and 6.0 eV for Ni and Ho atoms, respectively. The U value was set to 0 eV for Al atoms due to the lack of localized electrons. The spin–orbit coupling calculation was not performed due to high computing times; instead, a Koelling–Harmon relativistic scheme was used for faster calculation [36]. To calculate the electronic density of states (DOS), a 13 × 13 × 13 k-mesh generated by the Monkhorst–Pack scheme was used to integrate the Brillouin zone. For the plane-wave propagation along the crystal, a cut-off energy of 500 eV was applied [37,38,39,40]. The energy convergence criterion was set at 1 × 10^−6^ eV/atom for self-consistent field cycles. The maximum values of the convergence thresholds were 0.03 eV/Å, 0.05 GPa, and 0.001 Å for force, stress, and displacement, respectively. During the geometric optimization process, the compressive external stress was applied along the a, b, and c axes to consider the effect of external hydrostatic pressure using the Broyden–Fletcher–Goldfarb–Shanno (BFGS) algorithm. The Cauchy stress tensor was used as σ_ij_ = −P δ_ij_, where P and δ_ij_ are the hydrostatic pressure value and the Kronecker delta, respectively. The latter tensor is equivalent to hydrostatic pressure P. The obtained bulk modulus B was obtained by fitting the third-order Birch–Murnaghan state equation.

**Figure 1:**
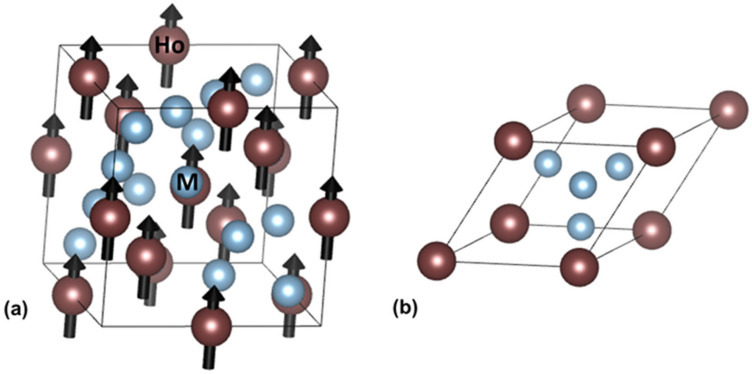
Schematic representation of the MgCu_2_ cubic crystal structure (**a**) and primitive cell (rhombohedral trigonal) (**b**) of the ferromagnetically ordered HoAl_2_ and HoNi_2_ Laves phases. The silver and brown spheres represent the elements Al or Ni and the rare-earth element Ho, respectively. The black arrows indicate that the Ho magnetic moment points along the *c*-axis.

The stoichiometric HoM_2_ with M = {Al, Ni} Laves phases crystallize in a cubic MgCu_2_-type structure with a space group Fd–3m. Figure 1 schematically shows the crystalline and magnetic structures and primitive cell of HoM_2_ (M = {Al, Ni}) Laves phases. In the AB_2_ structure, the A atom (i.e., Ho) occupies the 8a Wyckoff site at (0 0 0), while the B atoms (i.e., Al and Ni) occupy the 16d Wyckoff site at (5/8 5/8 5/8) positions. For Al, Ni, and Ho, the electronic configuration is described as [Ne] 3*s*^2^ 3*p*^1^, [Ne] 3*s*^2^ 3*p*^6^ 3*d*^8^ 4*s*^2^, and [Ne] 3*s*^2^ 3*p*^6^ 3*d*^10^ 4*s*^2^ 4*p*^6^ 4*d*^10^ 5*s*^2^ 5*p*^6^ 4*f*^11^ 6*s*^2^, respectively. It is important to note that for simulating HoAl_2_ and HoNi_2_ Laves phases, in the search for accuracy, we used experimental lattice parameters rather than those obtained from minimizing the total energy as a function of volume for the crystalline structures. The lattice parameters *a* = *b* = *c* were 7.810 Å for HoAl_2_ and 7.130 Å for HoNi_2_ [14,15]. The collinear ferromagnetic ordering of each compound was modeled assuming that only the rare-earth atoms, specifically Ho at 8a positions, possess a magnetic moment aligned along the <001> direction. Zero magnetic moment was assumed for Al and Ni atoms, which are located at 16a positions.

## 3. Results and Discussion

### 3.1. Electronic Properties

**Figure A1:**
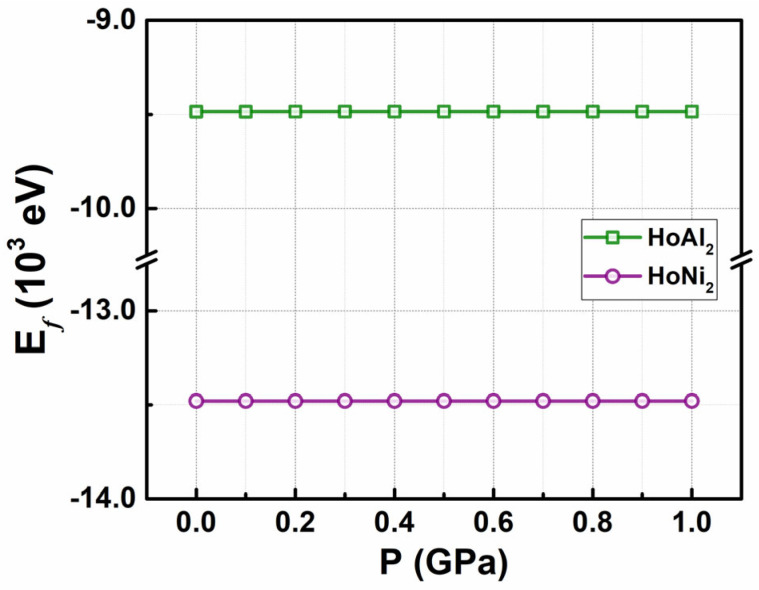
Calculated formation energy as a function of the applied hydrostatic pressure for ferromagnetically ordered HoAl_2_ and HoNi_2_ Laves phases.

Table 1 displays the lattice parameter and interatomic distances between Al-Al, Ho-Al, and Ho-Ni in the Laves phase HoM_2_ with M = {Al, Ni}, under applied hydrostatic pressures from 0 GPa to 1.0 GPa. It is worth noting that HoAl_2_ is more sensitive to external pressure than HoNi_2_ alloys. The structural stability of both HoAl_2_ and HoNi_2_ remains unchanged across the entire range of applied pressures. Their corresponding formation energy E*_f_* values at P = 0 GPa are −9.485 × 10^3^ eV (HoAl_2_) and –13.470 × 10^3^ eV (HoNi_2_). For non-zero pressures, the overall magnetic behaviors do not exhibit significant variation due to the minimal compaction in their crystal structures. All formation energy values remain nearly constant with a virtually negligible increase (less than 1%) under external pressures up to 1.0 GPa; details are shown in Figure A1 in the Appendix A Section. Additionally, substituting Al atoms with Ni atoms leads to a reduction in lattice parameters, resulting in an increase in bulk modulus at applied pressures around 1.0 GPa, as detailed in Table 1. The obtained bulk modulus B follows an increasing linear dependence with the applied pressure, having slopes dB/dP of 27.93 and 46.44 with intercepts B_0_ = 45.06 GPa and 49.41 GPa for HoAl_2_ and HoNi_2_, respectively. The R^2^ values of the fitting are 0.93 and 0.99 for each alloy. Finally, a measure of lattice constant changes can be deduced from the relative compressive stress ∆ caused by applied pressure; see Table 1. This quantity can be calculated as ∆ = (V_P_ − V_0_)/V_0_ × 100%, where ∆V is the volume difference calculated as V_P_ − V_0_. The resulting ∆(P = 1 GPa) values are −2.88% and −1.87% for HoAl_2_ and HoNi_2_, respectively.

### 3.2. Determination of Electronic Coefficient in Specific Heat Capacity

Considering that each atom donates one electron to the Fermi gas in the solid, the free-electron number densities (N/V) for HoAl_2_ and HoNi_2_ are 1.678 × 10^28^ m^−3^ and 2.203 × 10^28^ m^−3^, respectively. On the other hand, M(HoAl_2_) = 218.894 g/mol, ρ(HoAl_2_) = 6.08 g/cm^3^ [14], M(HoNi_2_) = 282.316 g/mol, and ρ(HoNi_2_) = 10.33 g/cm^3^ [41] are used.

Bearing in mind that in metals theory at P = 0 GPa and T = 0 K, the Fermi energy *E_F_* can be calculated as follows:

(1) $$E_{F}=\frac{h^{2}}{2\;m_{e}}{\left(\frac{3\;N}{8\;\pi \;V}\right)}^{2/3}$$

where h and $m_{e}$ are the Planck constant and the electron mass, respectively. Using Equation (1) and previous values, the Fermi temperature *T_F_* can be calculated through the following formulae:

(2) $$T_{F}=\frac{E_{F}}{k_{B}}$$

where $k_{B}$ is the Boltzmann constant. The electronic coefficient $\gamma_{e}$ of specific heat capacity can be calculated using the following equation:

(3) $$\gamma_{e}=\frac{\pi^{2}\;k_{B}\;N_{A}}{3\;T_{F}}$$

The calculated values of the Fermi energy and its temperature and the electronic coefficient $\gamma_{e}$ using Equations (1)–(3) are listed in Table 2. The *N*/*V*, *E_F_*_,_ and *T_F_* values agree with those obtained for other elements such as K, Ag, and Cu [42].

Another way to determine the electronic heat capacity coefficient $\gamma_{e}$ for alloys is through DFT quantum calculations. The Einstein–Debye model states that $c_{p}\left(T\right)=\gamma_{e}T+\beta_{ph}T^{3}$ at temperatures *T* << *T*_D_, where *T*_D_ is the Debye temperature, and the terms $\gamma_{e}T$ and $\beta_{ph}T^{3}$ represent the electronic and phonon contributions to the specific heat capacity, respectively. From the Sommerfeld approximation [43], the coefficient $\gamma_{e}$ can be calculated as follows:

(4) $$\gamma_{e}=\frac{\;\pi^{2}\;{k_{B}}^{2}}{3}\Delta n(E_{F})$$

where $\Delta n(E_{F})$ is the density of electrons per eV at the Fermi level. This expression applies when external hydrostatic pressure is considered.

**Table 2:** The calculated values of Fermi energy (*E_F_*), Fermi temperature (*T_F_*), and the electronic specific heat capacity coefficient ($\gamma_{e}$) obtained by Equations (1)–(3) for both Laves phases (HoAl_2_ and HoNi_2_). Other pure elements [42] and Laves phases RM_2_ with R = (La, Lu, Ho) and M = (Al, Ni) [44,45,46] are included for comparison purposes.

Laves Phases or Elements	*N*/*V* (×10^28^ m^−3^)	$E_{F}$ (eV)	$T_{F}$ (×10^4^ K)	$\gamma_{e}$ (×10^–3^ J mol^–1^ K^–2^)	Reference
HoAl_2_	1.678	2.392	2.777	0.984	this work
HoNi_2_	2.203	2.869	3.331	0.820	this work
K	1.40	2.13	2.47	1.106 ^a^	[42]
Ag	5.86	5.53	6.41	0.426 ^a^	[42]
Cu	8.47	7.06	8.19	0.333 ^a^	[42]
LaAl_2_	-	-	-	10.6 ^c^	[44]
LuAl_2_	-	-	-	5.5 ^c^	[44]
LaNi_2.2_	-	-	-	4.8 ^c^	[44]
LuNi_2_	-	-	-	4.6 ^c^	[44]
LuAl_2_^b^	-	-	-	5.4 ^c^	[45]
HoAl_2_ bulk polycrystalline	-	-	-	7.0 ^a^	[46]

^a^ calculated using Equation (3). ^b^ *c_p_*(T) data is like HoAl_2_. ^c^ determined from *c_p_*(T) data.

**Figure 2:**
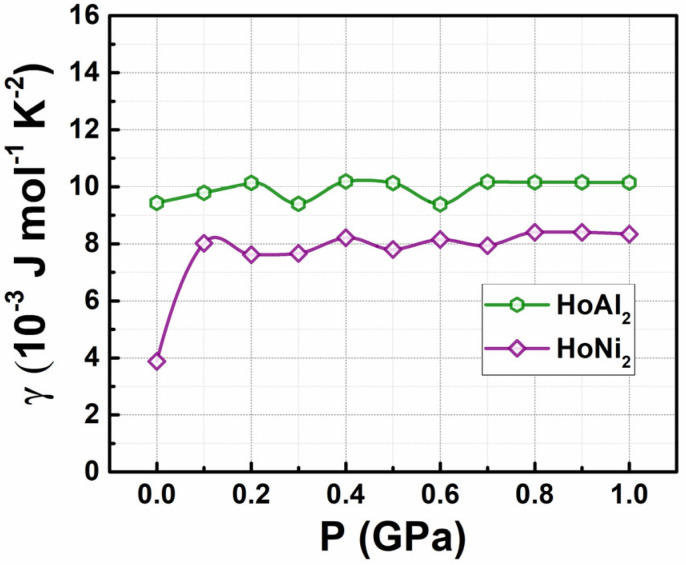
Electronic specific heat capacity coefficient as a function of the applied hydrostatic pressure for HoAl_2_ and HoNi_2_ alloys.

Figure 2 shows how the electronic specific heat capacity coefficients, calculated by Equation (4), change with increasing external hydrostatic pressures up to 1.0 GPa for the studied Laves phases. For HoAl_2_, the obtained $\gamma_{e}$ value at P = 0 GPa is 9.43 × 10^−3^ J mol^−1^ K^−2^. This result differs from that obtained through the electron gas model in metals (Table 2); the values tend to slightly increase, reaching an average of 10.02 × 10^−3^ J mol^−1^ K^−2^ as the applied pressure increases. Conversely, the initial value for the HoNi_2_ alloys at 0 GPa (i.e., 3.87 × 10^−3^ J mol^−1^ K^−2^) is like the value calculated using the gas model in metals; see Table 2 for details (0.820 × 10^−3^ J mol^−1^ K^−2^). Later, it increases noticeably to 8.02 × 10^−3^ J mol^−1^ K^−2^ with an applied pressure of 0.1 GPa and then remains nearly constant at an average value of 8.05 × 10^−3^ J mol^−1^ K^−2^. At non-zero pressures, the obtained values for HoAl_2_ and HoNi_2_ are closer to each other, as calculated by DFT+U modeling.

Von Ranke et al. reported electronic specific heat capacity coefficients of 10.6 × 10^−3^, 5.5 × 10^−3^, 4.8 × 10^−3^, and 4.6 × 10^−3^ J mol^−1^ K^−2^ for LaAl_2_, LuAl_2_, LaNi_2.2_, and LuNi_2_, respectively [44]. De Oliveira and colleagues reported a $\gamma_{e}$ experimental value of 5.4 × 10^−3^ J mol^−1^ K^−2^ from the *c_p_*(T) curve for non-ferromagnetic LuAl_2,_ stating that this alloy shows the same structure as HoAl_2_ and a similar $\gamma_{e}$ value [45]. Campoy et al. experimentally determined a value of $\gamma_{e}$ = 7.0 × 10^−3^ J mol^−1^ K^−2^ for a bulk polycrystalline HoAl_2_ alloy from *c_p_*(T) data [46]. The DFT and Fermi gas approach of $\gamma_{e}$ values at P = 0 GPa for the Laves phase alloys agree with experimental ones obtained by *c_p_*(T) data.

### 3.3. Electronic Density of States

#### 3.3.1. Total Density of Electronic States at P = 0 GPa

**Figure 3:**
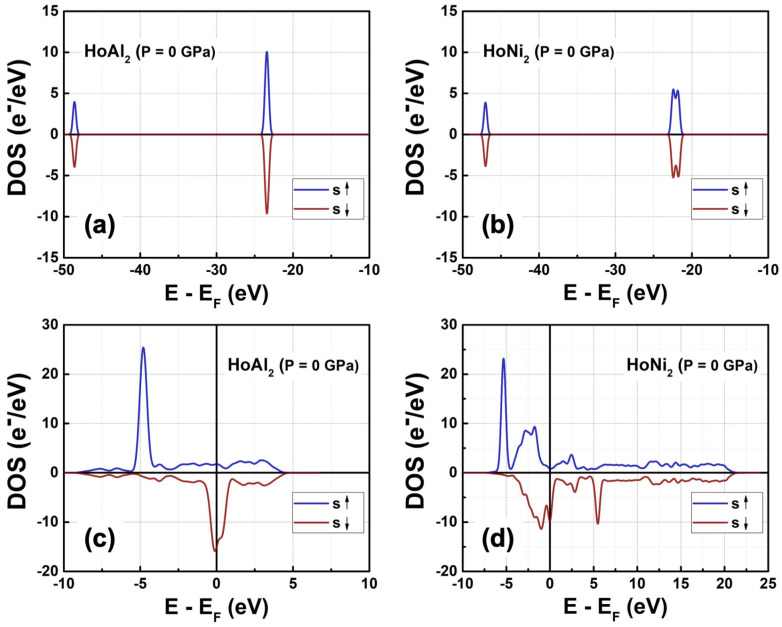
Total DOS at P = 0 GPa obtained for ferromagnetically ordered (**a**,**c**) HoAl_2_ and (**b**,**d**) HoNi_2_ compounds. The spin-polarized electronics bands are represented by (**a**,**b**) *s* and *p* orbitals, and (**b**,**d**) *d-f* hybridized orbitals.

Figure 3 shows the total density of electronic states (DOS) obtained in ferromagnetically ordered crystal structures HoM_2_ with M = {Al, Ni} Laves phases. The electronic structure for both compounds, *s* and *p* orbitals, is nearly symmetric and localized at deeper energies compared to *d* and *f* orbitals, which are at the Fermi level. The *s* orbitals are localized between −49.00 eV and −48.00 eV with a maximum electronic density of 3.92 e^−^/eV at −48.59 eV for HoAl_2_. When the post-transition metal (Al) is replaced by a transition metal (Ni), the localization of *s* orbitals shifts toward higher energies (i.e., between −47.43 eV and −46.19 eV) with a similar electronic density of 3.87 e^−^/eV. The *p* orbitals in HoAl_2_ are localized in the energy range of −24.30 eV ≤ *E*−*E_F_* ≤ −22.62 eV, while they are positioned between −23.00 eV and −21.32 eV for HoNi_2_. Our calculations show that the spin-up channel is shifted to lower energies compared to the spin-down channel while maintaining symmetry between both alloys. It is important to note that substituting Al with Ni reduces the maximum value in the DOS of *p* bands from 9.53 e^−^/eV to 5.95 e^−^/eV, and the DOS curve tends to flatten into a double peak; see Figure 3a,b.

The *d* and *f* orbitals are very close to the Fermi level. Specifically, *d* bands range from −5.2 eV to 10.0 eV, while *f* bands range from −5.3 eV to 3.0 eV. Both the DOS of *d* and *f* orbitals exhibit a notable asymmetry. Additionally, *f* bands are the most populated in both compounds. Therefore, the localized *f* electrons continue to be responsible for ferromagnetic order in HoM_2_ (M = {Al, Ni}) Laves phases. Replacing the post-transition metal Al with Ni leads to symmetry collapse of *d* orbitals, causing hybridization among *d* and *f* bands. This suggests that the magnetic behavior of HoAl_2_ arises from localized electrons in *f* orbitals, and both itinerant and localized electrons in *d* and *f* orbitals contribute to ferromagnetism in HoNi_2_.

The net magnetic moment [43] can be calculated using the following equation:

(5) $$\mu_{T}=\int_{E_{1}}^{E_{F}}n_{S↑}\left(E\right)dE\;-\int_{E_{2}}^{E_{F}}n_{S↓}\left(E\right)dE$$

where *E*_1_ and *E*_2_ represent the starting energies of electronic states for spin-up and spin-down channels, respectively. The total magnetic moments along the <001> *c*-axis obtained for ferromagnetically ordered HoAl_2_ and HoNi_2_ Laves phases are 8.61 µ_B_/f.u. and 8.12 µ_B_/f.u., respectively. These values, derived from DOS, match the single-crystalline data previously reported in scientific literature: 9.15 µ_B_/f.u. to 9.18 µ_B_/f.u. for HoAl_2_ [18,25] and 8.52 µ_B_/f.u. for HoNi_2_ [17]. Notice that the magnetic moment measurements for polycrystalline ribbons of HoAl_2_ [14] were carried out using in 9 T Dynacool PPMS-VSM magnetometer along the major axis of the ribbon length (in-plane). The *T*_C_ value is 29 K for this HoAl_2_ sample. As summarized in Table 3, the net magnetic moments determined for polycrystalline ribbons [14,15] and bulk/massive [47,48] samples are close to that obtained from DFT quantum calculations.

**Table 3:** Cell parameter *a*, calculated magnetic moment µ_T_, and magnetization of HoAl_2_ and HoNi_2_ compared with experimental data reported in the literature [14,15,16,17,22,47,48].

Laves Phase	Alloy Type	*a* (Å)	µ_T_ (µ_B_/f.u.)	$T_{C}$ (K)	M_S_ (Am^2^kg^−1^)	Magnetization Axis	Reference
HoAl_2_	DFT+U framework	7.810	8.61 ^a^	-	220 ^a^	<001>; intermediate	this work
	single-crystal	7.816 ^c^ 7.838	9.18 ^b^ 9.15 ^c^	31.5 29.0	235 ^b^ 234 ^c^	<011>; easy	[16] [22]
	bulk polycrystalline	7.8024	7.86	27.0	201	close to <001>; intermediate	[47]
	polycrystalline ribbons (20 m/s)	7.8109	7.08 ^c, e^	24.0	181 ^c, e^	close to <001>; intermediate	[14]
HoNi_2_	DFT+U framework	7.130	8.12 ^a^	-	161 ^a^	<001>; easy	this work
	single-crystal	-	8.52 ^d^	13.4	168 ^d^	<001>; easy	[17]
	bulk polycrystalline	7.1318	8.40	22.0	167	very close to <001>; easy	[47,48]
	polycrystalline ribbons (20 m/s)	7.1497	8.02 ^c^	13.9	159 ^c^	close to <001>; easy	[15]

**Figure 4:**
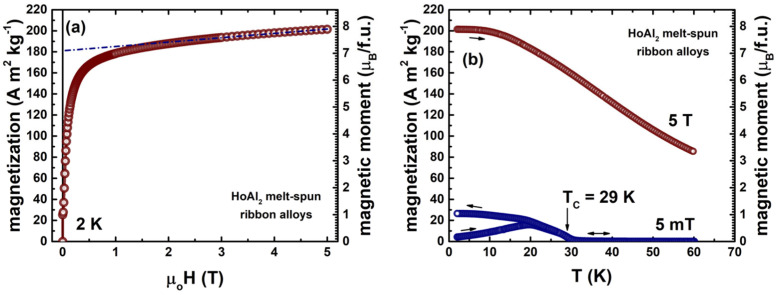
Magnetization isotherm at 2 K (**a**) and temperature dependence of magnetization measured under magnetic fields of 5 mT and 5 T (**b**) for HoAl_2_ melt-spun ribbons fabricated at 8 m/s in a melt-spinner. The short-dashed line in (**a**) indicates the saturation magnetization extrapolated to a zero magnetic field (reported in Table 3). The magnetic moment per formula unit is calculated from the magnetization values. As pointed out by the vertical arrow in (**b**), the sample shows *T*_C_ = 29 K. More experimental details can be found in reference [14], and the demagnetizing field effect was neglected when measuring along the large side of ribbon flakes.

Experimental data measured at the following temperatures: ^a^ T = 0 K. ^b^ T = 4.2 K. ^c^ T = 2.0 K. ^d^ T = 1.4 K. ^e^ determined from Figure 4. The experimental crystal structure cell unit is reported at room temperature.

#### 3.3.2. Total Electronic Density of States at 0 GPa < P ≤ 1.0 GPa

**Figure 5:**
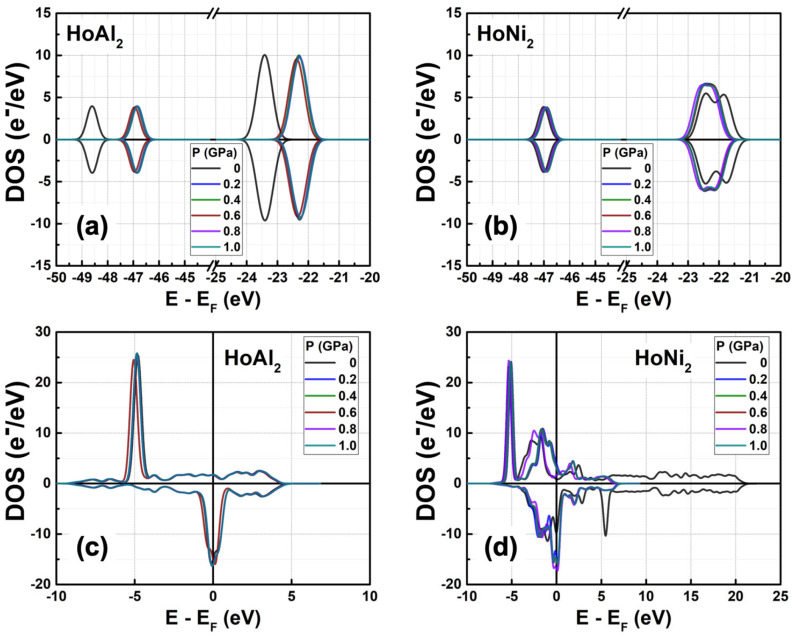
Total DOS obtained for ferromagnetically ordered (**a**,**c**) HoAl_2_ and (**b**,**d**) HoNi_2_ crystal structures under external hydrostatic pressures between 0 GPa and 1.0 GPa. Selected curves are shown to provide visual insight.

Figure 5 shows the calculated DOS for ferromagnetically ordered HoAl_2_ and HoNi_2_ Laves phases under external hydrostatic pressures from 0 GPa to 1.0 GPa. For both alloys, a small increase in the external pressure applied to the crystal structure causes a shift to higher energies of the *s* and *p* orbitals. The shift is more evident in HoAl_2_ (Figure 5a) than in HoNi_2_ (Figure 5b). Meanwhile, *d* and *f* orbitals move closer to the Fermi energy level. For HoNi_2_, a significant redistribution occurs in the electronic population of *f* orbitals at non-zero pressure; the maximum of the spin-up channel increases from 14.26 e^−^/eV to 23.48 e^−^/eV, while the initial splitting of the spin-down channel disappears, and a maximum electronic density of −14.35 e^−^/eV is observed.

**Figure 6:**
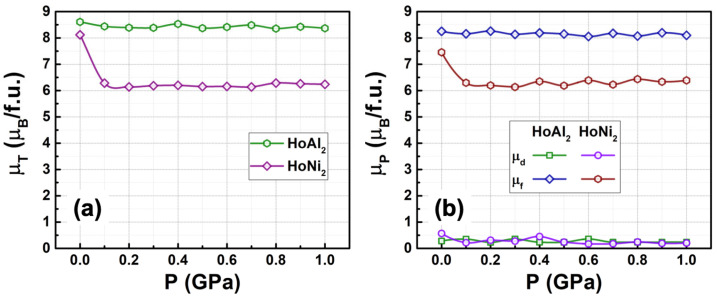
(**a**) Total magnetic moment and (**b**) contributions from electrons in the *d* and *f* orbitals to the magnetic moment in ferromagnetically ordered HoAl_2_ and HoNi_2_ Laves phases, plotted as a function of external hydrostatic pressure (0 GPa ≤ P ≤ 1.0 GPa). The μ_T_ and μ_P_ values are obtained from the total and partial DOS, respectively.

Figure 6a shows the calculated total magnetic moment versus hydrostatic pressures in the range of 0 GPa ≤ P ≤ 1.0 GPa for ferromagnetically ordered HoM_2_ (M = {Al, Ni}) crystal structures. For HoAl_2_, the total magnetic moment remains nearly constant (around a mean value of 8.49 µ_B_/f.u.) as the applied pressure increases up to 1 GPa. In contrast, for HoNi_2_, the total magnetic moment at P = 0 GPa (8.12 µ_B_/f.u.) decreases to 6.29 µ_B_/f.u. at 0.1 GPa and then remains nearly constant for a non-zero applied pressure. This 22.5% decrease indicates that DOS is modified after 0.1 GPa, which can be useful in a multicaloric approach during magnetic phase transition [2,3].

Additionally, Figure 6b displays the calculated magnetic moment associated with *d* and *f* orbitals for both alloys. It is important to note that the magnetic order mainly results from localized *f* electrons. The itinerant electrons contribute only minimally to the magnetic moment. Moreover, for HoNi_2_, the magnetic contribution of 4*f* electrons decreases from 7.45 µ_B_/f.u. to 6.31 µ_B_/f.u. under an applied pressure of 0.1 GPa and then remains nearly constant at a non-zero pressure value.

#### 3.3.3. Electronic Partial Density of States at P = 0 GPa

**Figure 7:**
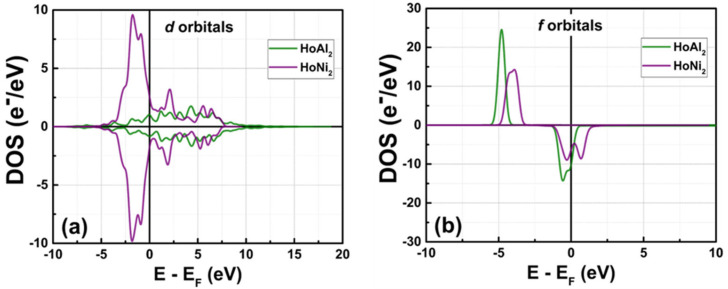
Partial DOS at P = 0 GPa obtained for (**a**) *d* and (**b**) *f* orbitals in the ferromagnetically ordered HoM_2_ with M = {Al, Ni} Laves phases.

**Figure A2:**
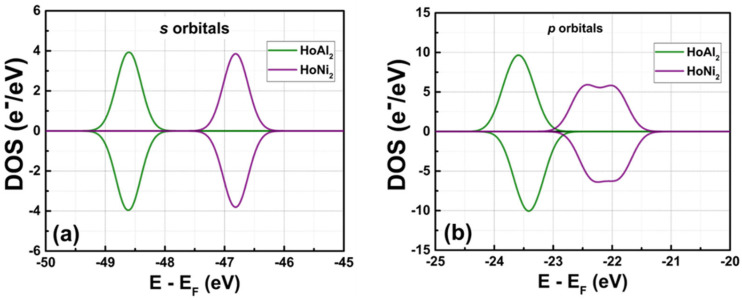
Partial electronic density of states at P = 0 GPa calculated for (**a**) *s* and (**b**) *p* orbitals in ferromagnetically ordered HoAl_2_ and HoNi_2_ Laves phases.

The obtained partial density of electronic states (PDOS) corresponding to *d* and *f* orbitals is shown in Figure 7. Once a transition metal like Ni replaces the post-transition metal Al in the crystalline structure, the electronic population of 3*d* electrons increases, while the density of electrons in 4*f* orbitals decreases, resulting in a broadened peak of the DOS curve. The contributions per orbital to the total magnetic moment are listed in Table 4. At P = 0 GPa, electrons localized at *d* orbitals contributed less to the net magnetic moment in the crystal structure HoAl_2_ (i.e., 0.28 µ_B_/f.u.) compared to HoNi_2_ (i.e., 0.57 µ_B_/f.u.). Therefore, the magnetic behavior of the ferromagnetically ordered HoAl_2_ crystal structure is just due to unpaired electrons localized at *f* orbitals. When Al is fully replaced by Ni, the number of unpaired electrons in the *d* orbital increases; as a result, the magnetic moment of HoNi_2_ arises from 3*d* and 4*f* electrons. Moreover, *s* and *p* orbitals shift to higher energies when Al is replaced by Ni, and *p* orbitals tend to decrease their maximum population, resulting in a broadened and flattened peak band. Partial DOS for *s* and *p* orbitals is shown in Figure A2. Finally, the contribution of electrons localized at *s* and *p* orbitals to the total magnetic moment is almost negligible for both HoAl_2_ and HoNi_2_.

On the one hand, Figure 7a shows that the 4*d* and 3*d* electrons of Ho in HoAl_2_ are located very close to the Fermi level with a very low density of states. In contrast, the 4*f* electrons of Ho in the spin-up channel are situated at −4.77 eV, far from the Fermi level, while the 4*f* electrons in the spin-down channel are practically on the Fermi energy level (at −0.70 eV); see Figure 7b. For HoNi_2_, the 3*d* electrons in both spin-up and spin-down channels are near the Fermi level (at −1.75 eV). Conversely, the 4*f* electrons of Ho at the spin-up channel are localized at −4.03 eV, far away from the Fermi level; in the case of the spin-down channel, 4*f* electrons are very close to the Fermi level (from −1.16 eV to 0 eV). Notice that the amount of Ho 4*f* electrons in HoAl_2_ has larger peaks in the density of states compared to the broad, lower peaks of Ho 4*f* electrons in HoNi_2_. Finally, the Ni 3*d* electrons have a higher density of states compared to the Ho 4*d* and 3*d* electrons.

Table 4 presents the calculated net magnetic moment computed from the simulated DOS at P = 0 GPa for ferromagnetically ordered HoAl_2_ and HoNi_2_ Laves phases, along with the contribution of electrons at *s*, *p*, *d*, and *f* orbitals to the total magnetic moment. When Al is fully replaced by Ni, the contribution of *d* electrons to the total magnetic moment slightly increases. Furthermore, the main contributor to magnetic behavior in ferromagnetically ordered HoM_2_ with M = {Al, Ni} is the *f* electrons.

#### 3.3.4. Electronic Partial Density of States at 0 GPa < P ≤ 1.0 GPa

**Figure A3:**
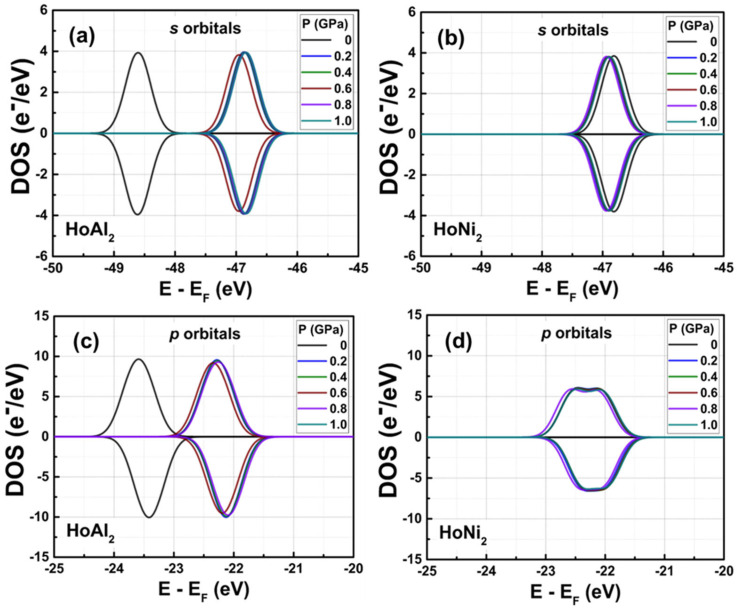
Partial electronic density of states calculated for *s* and *p* orbitals in ferromagnetically ordered HoAl_2_ (**a**,**c**) and HoNi_2_ (**b**,**d**) Laves phases within the pressure range of 0 GPa ≤ P ≤ 1.0 GPa.

For ferromagnetically ordered HoM_2_ (M = {Al, Ni}) Laves phases, the applied external hydrostatic pressure induces a shift in the energies of the *s*, *p*, *d*, and *f* bands. This shift is more noticeable for the *s* and *p* bands in HoAl_2_ than in HoNi_2_. The PDOS obtained for *s* and *p* orbitals are shown in Figure A3 of the Appendix A Section.

**Figure 8:**
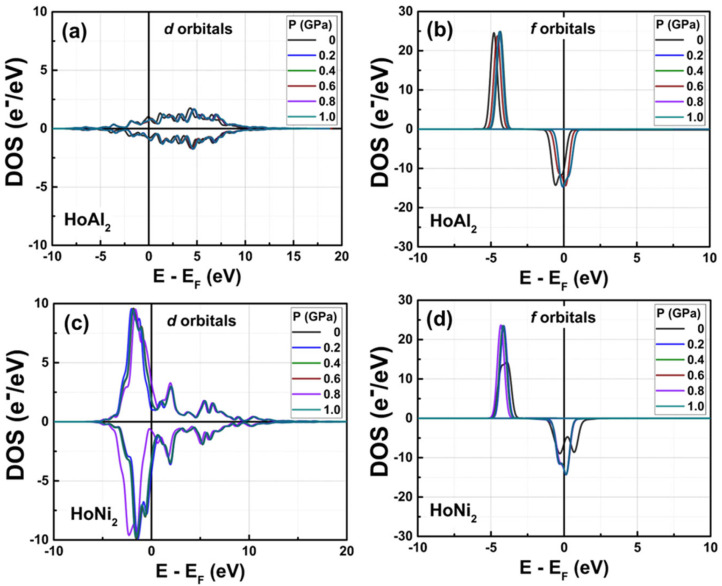
Partial DOS obtained for (**a**,**c**) *d* and (**b**,**d**) *f* orbitals of ferromagnetically ordered HoAl_2_ and HoNi_2_ crystal structures, respectively, under external hydrostatic pressures ranging from 0 GPa to 1.0 GPa (selected curves are shown for better visualization).

Figure 8 displays the PDOS for the *d* and *f* orbitals under external hydrostatic pressures ranging from 0 GPa to 1.0 GPa. In the ferromagnetically ordered HoAl_2_ Laves phase, the applied pressures (0 GPa to 1.0 GPa) cause a slight rearrangement of the 3*d* and 4*d* Ho spin-up and spin-down channel bands; see details in Figure 8a. The 4*f* electrons of Ho in the spin-up channel, initially localized far from the Fermi level at −4.77 eV, move slightly closer to the Fermi level as pressure increases, reaching −4.34 eV at 1.0 GPa (Figure 8b). Meanwhile, the 4*f* electrons in the spin-down channel, initially localized at −0.70 eV, nearly reach the Fermi level.

Figure 8c,d illustrate that for HoNi_2_, the 3*d* electrons of Ni in both spin-up and spin-down channels do not undergo significant changes as the external pressure increases from 0 GPa to 1.0 GPa. Furthermore, the 4*f* electrons of Ho, a rare-earth element, in both spin-up and spin-down channels show an increase in their maximum electronic density under non-zero applied pressure. It is important to note that the initial splitting of the spin-down channel tends to disappear. External pressure causes a redistribution of the electronic population across the orbitals for both alloys, but the most affected orbitals are the 4*f* orbitals of HoNi_2_. The maximum population of electrons with spin-up at *f* orbitals of HoNi_2_ increases from 14.26 e^−^/eV to 23.48 e^−^/eV, and their electronic density of the spin-down channel rises to −14.35 e^−^/eV near the *E_F_* level, as shown in Figure 8d.

## 4. Conclusions

This study examined how hydrostatic pressures from 0 GPa to 1.0 GPa affect the crystal stability, electronic properties, and magnetic properties of ferromagnetically ordered HoAl_2_ and HoNi_2_ Laves phases through DFT+U calculations using the RPBE exchange-correlation functional within the GGA framework. All calculations were performed along the <001> direction, and the net magnetic moment obtained remains nearly constant for ferromagnetically ordered HoAl_2_, with values of 8.61 µ_B_ per formula unit along the intermediate magnetization axis. The ferromagnetically ordered HoNi_2_ Laves phases experience a reduction in their initial magnetic moment from 8.12 µ_B_/f.u. to 6.29 µ_B_/f.u. in the easy magnetization axis when subjected to non-zero applied pressure. The latter HoNi_2_ Laves phase is useful for the multicaloric approach in solid-state cooling by using magnetocaloric and barocaloric effects. The magnetic moment can be modified by 22.5% under 0.1 GPa in the range of fully reversible loading and unloading regimes in the alloy when the hydrostatic pressure is applied and released, respectively. The HoAl_2_ Laves phase is the most affected by the applied pressures, with a compressive stress of −2.88%, while the HoNi_2_ exhibits a compressive stress of −1.87%. The interatomic distances change very little within the pressure range studied. The ferromagnetic order persists, displaying a reorganization of the electronic states, with the *f* orbitals of HoNi_2_ Laves phases being the most affected. The stability of the ferromagnetically ordered HoAl_2_ and HoNi_2_ Laves phases’ crystal structures remains unaffected across the entire range of applied pressures, and the formation energy stays constant up to 1.0 GPa.

## Figures and Tables

**Table 1:** Diagonal component σ_ij_ of the stress tensor, bulk modulus B, primitive unit cell volume V_P_ (i.e., rhombohedral trigonal), compressive stress Δ, and interatomic Al-Al, Ho-Al, and Ho-Ni distances obtained for the HoAl_2_ and HoNi_2_ Laves phases under applied hydrostatic pressures of 0 GPa ≤ P ≤ 1.0 GPa.

Alloy	HoAl_2_							HoNi_2_						
P (GPa)	B (GPa)	σ_ij_ (GPa)	a (Å)	d_Al-Al_ (Å)	d_Ho-Al_ (Å)	V_P_ (Å^3^)	Δ (%)	B (GPa)	σ_ij_ (GPa)	a (Å)	d_Ni-Ni_ (Å)	d_Ho-Ni_ (Å)	V_P_ (Å^3^)	Δ (%)
0.0	40.66	−0.00005	5.652	2.826	3.314	127.675	0.000	49.79	−0.00115	5.262	2.631	3.086	103.066	0.000
0.1	45.68	−0.10067	5.646	2.823	3.311	127.299	−0.294	56.58	−0.10030	5.258	2.629	3.083	102.840	−0.219
0.2	52.56	−0.19968	5.640	2.820	3.307	126.908	−0.600	58.98	−0.19724	5.255	2.628	3.081	102.655	−0.399
0.3	56.69	−0.29771	5.635	2.818	3.304	126.533	−0.894	60.33	−0.30162	5.252	2.626	3.080	102.480	−0.569
0.4	58.00	−0.39932	5.629	2.815	3.301	126.165	−1.182	68.79	−0.40011	5.248	2.624	3.077	102.241	−0.801
0.5	60.58	−0.49793	5.623	2.812	3.297	125.780	−1.484	71.50	−0.50638	5.245	2.623	3.076	102.072	−0.964
0.6	64.07	−0.60178	5.618	2.809	3.294	125.380	−1.797	76.23	−0.60287	5.242	2.621	3.074	101.868	−1.162
0.7	65.22	−0.70149	5.613	2.807	3.291	125.055	−2.052	82.56	−0.69933	5.239	2.620	3.072	101.703	−1.322
0.8	66.65	−0.80276	5.607	2.804	3.288	124.681	−2.345	84.36	−0.80034	5.235	2.618	3.070	101.479	−1.540
0.9	68.37	−0.89994	5.602	2.801	3.285	124.328	−2.621	91.47	−0.89917	5.232	2.616	3.068	101.321	−1.693
1.0	70.89	−0.99911	5.597	2.799	3.282	123.992	−2.884	98.45	−0.99998	5.229	2.615	3.066	101.132	−1.876

**Table 4:** Net magnetic moment values calculated using Equation (5) from the obtained DOS at P = 0 GPa and compared with experimental data [16,17]. Electronic quantities with spin up and down, along with the difference in electronic states at the Fermi level, are shown for the ferromagnetically ordered HoAl_2_ and HoNi_2_ Laves phases along the <001> direction.

Physical Magnitude	Alloy	
	HoAl_2_	HoNi_2_
n_S↑_ (*E_F_*) (e^−^/eV)	1.76	14.59
n_S↓_ (*E_F_*) (e^−^/eV)	−15.10	−9.11
Δn (*E_F_*) (e^−^/eV)	−13.34	5.48
$\mu_{S↑}$ (µ_B_/f.u.)	31.30	37.08
$\mu_{S↓}$ (µ_B_/f.u.)	−22.69	−28.96
$\mu_{T}^{DFT+U}$ (µ_B_/f.u.)	8.61	8.12
$\mu_{T}^{Exp}$ (µ_B_/f.u.)	9.18 ^†^	8.52 ^‡^
μ_s_ (μ_B_/f.u.)	−0.03	0.11
μ_p_ (μ_B_/f.u.)	0.04	−0.17
μ_d_ (μ_B_/f.u.)	0.28	0.57
μ_f_ (μ_B_/f.u.)	8.79	7.45
μ_P_ (μ_B_/f.u.)	9.08	7.96

Experimental magnetic moment values for single crystals are measured in ^†^ ref [16] and ^‡^ ref [17].

## Data Availability

The original contributions presented in this study are included in the article. Further inquiries can be directed to the corresponding author.
